# Effect of Acupuncture along Meridians on Pain Degree and Treatment of Acute Lumbar Sprain

**DOI:** 10.1155/2022/5497805

**Published:** 2022-07-23

**Authors:** Yi Li, Xiaokai Chen, Lvping Li, Feixiang Zeng, Juntao Li, Lan Lu

**Affiliations:** Rehabilitation Medicine Center, Huizhou Third People's Hospital, Guangzhou Medical University, No. 176, Dongping Section, Huizhou Avenue, Guangzhou, Guangdong 516002, China

## Abstract

**Aim:**

This study is aimed at investigating the effect of acupuncture along meridians on pain degree and treatment of acute lumbar sprain.

**Methods:**

A total of 96 patients with acute lumbar sprain from May 2019 to March 2021 in our hospital were selected and divided into the study and control groups. The patients in the control group were administered conventional western medicine and massage therapy, while the study group underwent acupuncture along meridians based on the control group. The therapeutic effect, visual analogue scale (VAS), Roland-Morris Disability Questionnaire (RMDQ), and lumbar range of motion (ROM) scores, emG inversion times, emG amplitude of the sacrospinalis muscle, and the serum TNF-*α* and IL-6 levels were determined.

**Results:**

The total effective rate of the study group was significantly higher than that of the control group. After treatment, the VAS, RMDQ, and ROM scores of the study group were significantly lower than those of the control group. Before the intervention, the EMG inversion times and the EMG amplitude of the spinous process muscle in the study group were not significantly different from those in the control group. After the intervention, the number and amplitude of EMG reversal in the study group were significantly higher than those in the control group. After the intervention, the serum levels of TNF-*α* (pg/ml) and IL-6 (pg/ml) in the study group were significantly lower than those in the control group. *Conclusion*. Meridian acupuncture for acute lumbar sprain can effectively improve body function, relieve pain, regulate serum inflammatory factors, and improve the overall therapeutic effect.

## 1. Introduction

Acute lumbar sprain is a clinically frequent type of disease, which mainly occurs in patients with acute lumbar soft tissue injury due to improper force during exercise or childbirth. Patients often experience different degrees of low back pain and activity limitation, which greatly threatens the patient's physical and mental health and quality of life [1–3]. If the acute lumbar sprain is not intervened in a timely and effective manner, it is likely to turn into a long-term chronic lumbar sprain, increasing the difficulty of treatment [4–6]. Therefore, methods of conducting safe and effective intervention for patients with acute lumbar sprain are still a hot clinical issue.

Currently, for patients with acute lumbar sprain, their treatments are physical therapy, oral analgesics, and bed rest. However, there are obvious adverse reactions, slow starting effects, and other deficiencies, making it difficult to achieve the ideal effect [7]. Traditional Chinese medicine has a unique understanding and insight, and there are many methods for treating acute lumbar sprain, including cupping, massage, acupuncture, and internal and external application of traditional Chinese medicine, among which acupuncture is widely used [8]. However, scholars such as Ren et al. [9] pointed out that the conventional acupuncture treatment of waist acupoint has obvious limitations, and patients with acute lumbar sprain have difficulty bending at will to cooperate with the acupuncture therapy in the early stages of the disease. A possible solution is to acupuncture along the meridians, the meridians follow the meridians to the location of the pain point, and select the easier distal acupoints, which can effectively relieve the pain and reduce the patient's limited mobility.

In this study, 96 patients with acute lumbar sprain in our hospital were selected and divided into a study group and a control group to explore the effect of meridian acupuncture on the pain degree and treatment of acute lumbar sprain.

## 2. Material and Methods

### 2.1. Baseline Data

This study was approved by the Ethics Committee of our hospital. A total of 96 patients with acute lumbar sprain from May 2019 to March 2021 in our hospital were selected and divided into the study and control groups according to the simple random number table method, with each group having 48 patients. There were 25 males and 23 females in the study group. The average age was 43.59 ± 10.79 years, ranging from 22 to 65 years; the duration of disease ranged from 1 to 6 days, with an average of 3.48 ± 2.02 days; and the body mass index (BMI) was 17.8 ~ 24.9 kg/m^2^, with an average of 21.39 ± 2.18 kg/m^2^. There were 27 males and 21 females in the study group. The average age was 45.11 ± 12.13 years, ranging from 21 to 69 years; the duration of disease ranged from 1 to 6 days, with an average of 3.60 ± 1.97 days; and the body mass index (BMI) was 17.4-25.8 kg/m^2^, with an average of 22.01 ± 2.05 kg/m^2^. The clinical data of sex, age, course of disease, and BMI were balanced and comparable between the two groups (*P* > 0.05).

### 2.2. Selection Criteria

#### 2.2.1. Inclusion Criteria

Inclusion criteria are as follows: (1) patients diagnosed by comprehensive imaging examination, laboratory examination, medical history, and clinical symptoms; (2) patients without ligament rupture, dislocation, fracture; (3) age > 18 years old; (4) awareness of this study and signature of patients with informed consent; and (5) the patients have good compliance and can cooperate with the completion of the investigation.

#### 2.2.2. Exclusion Criteria

Exclusion criteria are as follows: (1) women who are breastfeeding and pregnant; (2) patients with lumbar disc herniation or lumbar fracture; (3) patients with lumbar tuberculosis or tumor; (4) patients with organic diseases such as kidney and liver; (5) before the study, patients who have received antibiotics, hormones, immune, and anticoagulation therapy within one month; (6) patients with blood and immune system diseases; (7) patients with local skin ulcers or infections that cannot be treated with acupuncture and moxibustion; and (8) patients with mental system diseases.

### 2.3. Methods

The control group was administered conventional western medicine and massage therapy. Points were selected from the Yaojiaji, Dachang Shu, Chengshan, Huantiao, Yaoyangguan, Shenshu, and Ashi points. Treatment is performed by a trained and experienced acupuncturists.

Patients were guided to take the prone position and the operator stood on the affected side. Kneading started from top to bottom, the infiltration method was performed to press the Shenshu and the surrounding points, the thumbs were rubbed together and used to press the Ashi point, and gravity plucking was conducted through the elbow for the nodular and muscle spasm parts. Each part was operated on for at least 1 min with a focus on pressing the Shenshu point, and finally, both hands from top to bottom of the empty palm was used to beat the waist for 30 minutes per time. Aspirin was taken orally at 0.3 g once per day, and ibuprofen was taken orally at 0.3 g each time for two times a day for 10 days.

The study group underwent acupuncture along meridians based on the control group. If the pain site of the patient was in the middle of the spine, then the Du meridian was taken from the Renzhong point on the face. If the pain site was on one sides of the spine, then the same side of the Cuanzhu point on the face was selected in the Taiyang Bladder meridian, and the Houxi point, Yanglao point on the hand were also selected in the corresponding Taiyang Small Intestine meridian. The skin of the selected acupoints was disinfected, and a acupuncture needle (0.4 mm × 40 mm) was inserted with a depth of about 10 ~ 20 mm. Twisting was performed to increase the stimulus intensity after Qi was obtained. The patients were asked whether there was a sense of acid swelling. If there is a strong feeling of soreness, the needles were stopped, the waist was repositioned, the pain was pressed, and the needles were kept for 25 min. The intervention lasted 10 days.

### 2.4. Observation Indicators

Observation indicators are as follows: (1) Statistically, the treatment effect of the two groups showed that the lumbar movement was normal without limitations and the pain disappeared. Lumbar motion improved, there was a slight improvement, the pain was significantly relieved to improve. Failure to meet the above standards shall be invalid; (improved + cured)/total cases × 100% = total effective rate. (2) The visual analogue scale (VAS), Roland-Morris Disability Questionnaire (RMDQ), and lumbar range of motion (ROM) scores of the two groups before and after intervention were statistically analyzed. The VAS score ranges from 0 to 10 points, with a higher score associating with stronger pain. The RMDQ scale includes sleep, bending over, and walking, and the score ranges from 0 to approximately 24, where a lower score is associated with a better effect. The ROM scores are 0 (free bending and can bend to touch the ground starting from a standing position), 1 (bending to touch the knees), 2 (bending amplitude of <70°), 3 (barely able to bend), 4 (unable to bend), and 5 (associated with a stiff waist). (3) The number of emG reversals and the emG amplitude of the sacrospininous muscles before and after intervention were measured by Myo Move-COW surface emG. (4) The levels of serum inflammatory factors such as tumor necrosis factor-*α* (TNF-*α*) and interleukin-6 (IL-6) before and after intervention were measured by ELISA kits (TNF-*α*, PT518; IL-6, PI330; Beyotime, Beijing, China).

### 2.5. Statistical Method

The statistical analysis was performed by using SPSS version 22.0 (IBM SPSS Statistics, Armonk, NY, USA). The data were presented as $\bar{x}\pm s$, and *t*-test and enumeration data were expressed as *n* (%) and *χ*^2^ test. One-tailed *P* value less than 0.05 was considered statistically significant.

## 3. Results

### 3.1. Comparison of Treatment Effect between the Two Groups

In this study, all 96 patients with acute lumbar sprain from May 2019 to March 2021 in our hospital meet the inclusion criteria, and no subject was excluded from the current study. The total effectivity rate of the study group was higher than that of the control group (95.83% vs. 83.33%) (*P* < 0.05) (Table 1).

### 3.2. The Comparison of VAS, RMDQ, and ROM Scores between the Two Groups

There were no significant differences in the VAS, RMDQ, and ROM scores between the study and control groups (6.69 ± 1.07 vs. 6.83 ± 1.11, 13.24 ± 1.68 vs. 12.95 ± 1.81, and 3.28 ± 0.53 vs. 3.35 ± 0.60, respectively) before intervention (*P* > 0.05). After intervention, the scores in the study group were lower than those in the control group (1.04 ± 0.28 vs. 1.59 ± 0.37, 1.92 ± 0.41 vs. 3.15 ± 0.62, and 0.84 ± 0.19 vs. 1.24 ± 0.30, respectively) (*P* < 0.05) (Table 2).

### 3.3. Comparison of emG Inversion Times and emG Amplitude of the Sacrospinalis Muscle between the Two Groups

Before intervention, there were no significant differences in the number of emG inversion (times/s) and the emG amplitude (uV) of the saspinous muscle between the study and control groups (348.35 ± 51.69 vs. 352.07 ± 49.56 and 67.91 ± 13.64 vs. 70.05 ± 15.19, respectively) (*P* > 0.05). After intervention, the number of emG reversal (times/s) and the amplitude (uV) of the saspinous muscle in the study group were higher than those in the control group (630.01 ± 86.68 vs. 579.22 ± 71.04 and 130.11 ± 26.38 vs. 104.65 ± 19.82, respectively) (*P* < 0.05) (Table 3).

### 3.4. Comparison of Serum Inflammatory Factor Levels between the Two Groups

There was no significant difference in serum TNF-*α* (pg/ml) and IL-6 (pg/ml) between the study and control groups (27.91 ± 7.97 vs. 30.02 ± 8.19 and 22.39 ± 5.79 vs. 24.11 ± 6.38, respectively) before intervention (*P* > 0.05). After intervention, the levels in the study group were lower than those in the control group (10.22 ± 3.69 vs. 16.81 ± 4.15 and 9.71 ± 1.69 vs. 12.51 ± 2.96, respectively) (*P* < 0.05) (Table 4).

## 4. Discussion

Treatment is performed by two trained and experienced acupuncturists. Acute lumbar sprain can cause stretch and tear of lumbar muscles, peripheral ligaments, fascia, and other soft tissues, leading to aseptic inflammation, or excessive involvement of the posterior joint capsule and synovium due to changes in the anatomical position of the posterior lumbar joint, resulting in varying degrees of low back pain [10, 11]. Acute lumbar sprain has significant influence on the daily work and life of patients; therefore, it is of great importance to provide effective treatment as early as possible [12].

Ibuprofen and aspirin are often used in clinical western medicine as an intervention for patients with acute lumbar sprain, which can effectively adjust the state of lumbar microcirculation, reduce the degree of pain, and improve lumbar function; however, it is difficult to eliminate the fundamental induction of pain, and individual differences are large, making it difficult to achieve good results [13, 14]. In recent years, the application value of traditional Chinese medicine in acute lumbar sprain has attracted widespread attention. Traditional Chinese medicine classifies acute lumbar sprain as “Bi Zheng” and “Jin Shang,” which are mainly caused by fluttering, falling, and waist injuries. After injury, the lumbar meridians are blocked by blood stasis and Qi stagnation, which makes it difficult to nourish the lumbar spine, and the blood Qi is blocked and painful. Over time, it can cause the blood Qi stagnation to block the lumbar vein, and causes chronic low back pain. Therefore, the treatment of the disease should focus on promoting Qi and relieving pain, promoting blood circulation and removing blood stasis, and dredging meridians and activating collaterals by removing qi and blood stasis [15–17]. Meridian acupuncture is a soft tissue pain intervention method. Mainly based on the theory of meridians and collaterals in traditional Chinese medicine, this treatment effectively combines acupoint stimulation and physical therapy. By stimulating a distant acupoint along the meridian, it can transport the long-distance Qi and blood to the affected area for local circulation, increase the range of waist motion, and relieve pain [18].

For routine intervention, acupuncture along meridians was used to treat acute lumbar sprain. The results showed that the total effective rate of the study group was higher than that of the control group (95.83% vs. 83.33%), and the scores of the VAS, RMDQ, and ROM were lower than those of the control group (*P* < 0.05), indicating that acupuncture along meridians can effectively alleviate the pain of patients with acute lumbar sprain, restore the lumbar mobility and functional status, and improve the overall intervention effect. The reasons may be because most of the acupuncture points selected by the method of acupuncture along meridians are located at the distal ends of the head, face, or limbs. These acupuncture points are typically easy to “De Qi” (has a strong needle feeling, which is a simple and convenient operation), and can be used for emergency treatment. They are suitable for acute lumbar sprain where activities are inconvenient and associated with severe pain. In the treatment of acupuncture along meridians for patients with pain in the middle of the waist, acupuncture at the Renzhong acupoint, where the Yang and Yin meridians meet, can generate such intense stimulation and assist waist movement to eliminate pain. For patients with pain on each sides of the waist, Cuanzhu acupoint (also known as Shiguang acupoint in the Bladder meridian) is mainly used to transform the Yin Qi into Yang Qi, which can warm Yang Qi to clear the cold-dampness obstruction and dehumidifies to relieve pain. In addition, the Houxi acupoint (the intersection of the Small Intestine meridian and the Du meridian) cooperates with Yanglao acupoint, can also replenish the Yang Qi of the body, and enhances the needling response to relieve pain. Through the immediate effect of acupuncture, the local morbid blood stasis and pathogenic factors were dissipated. In the end, the Yang Qi warms Yin Qi , and balance is able to be achieved at the waist, so the pain of the patient can be quickly relieved.

Patients with acute lumbar sprain are often accompanied by varying degrees of muscle endurance decline and lumbar muscle spasm. Electromyography examination showed that the amplitude of muscle wave in the damaged part decreases and the number of electromyographic reversals decreases, resulting in reduced muscle contraction time and increases the risk of muscle fiber breakage [19, 20]. In this study, the number of myoelectric reversals and the amplitude of sacral spinous muscle wave in the study group after treatment were higher than those in the control group (*P* < 0.05), indicating that routine intervention combined with acupuncture along meridians can also significantly alleviate lumbar muscle spasm and promote the recovery of muscle tissue. Meanwhile, after lumbar muscle injury, the local inflammatory response was activated, which could produce a large number of inflammatory cytokines and aggravate the degree of local tissue injury. The results of this study showed that after the intervention, the serum level of TNF-*α* and IL-6 in the study group were lower than in the control group (*P* < 0.05).

## 5. Conclusion

In conclusion, routine intervention of meridian acupuncture in the treatment of acute lumbar sprain can effectively improve body function, relieve pain, regulate the level of serum inflammatory factors, and improve the overall therapeutic effect.

## Figures and Tables

**Table 1:** The comparison of treatment effect between the two groups (*n* (%)).

Groups	Cases	Cure	Improvement	Invalidation	Total effective rate
The study group	48	33 (68.75)	13 (27.08)	2 (4.17)	46 (95.83)
The control group	48	26 (54.17)	14 (29.17)	8 (16.67)	40 (83.33)
*χ* ^2^ value					4.019
*P* value					0.045

**Table 2:** Comparison of VAS, RMDQ, and ROM scores between the two groups ($\bar{x}\pm s$, points).

Groups	Cases	ROM		RMDQ		VAS	
		Before intervention	After intervention	Before intervention	After intervention	Before intervention	After intervention
The study group	48	3.28 ± 0.53	0.84 ± 0.19	13.24 ± 1.68	1.92 ± 0.41	6.69 ± 1.07	1.04 ± 0.28
The control group	48	3.35 ± 0.60	1.24 ± 0.30	12.95 ± 1.81	3.15 ± 0.62	6.83 ± 1.11	1.59 ± 0.37
*t* value		0.606	7.804	1.262	11.465	0.629	8.212
*P* value		0.546	0.000	0.210	0.000	0.531	0.000

**Table 3:** Comparison of emG inversion times and emG amplitude of the sacrospinalis muscle between the two groups ($\bar{x}\pm s$).

Groups	Cases	emG inversion times (times/s)		emG amplitude of sacrospinalis muscle (uV)	
		Before intervention	After intervention	Before intervention	After intervention
The study group	48	348.35 ± 51.69	630.01 ± 86.68	67.91 ± 13.64	130.11 ± 26.38
The control group	48	352.07 ± 49.56	579.22 ± 71.04	70.05 ± 15.19	104.65 ± 19.82
*t* value		0.360	3.140	0.726	5.346
*P* value		0.720	0.002	0.470	0.000

**Table 4:** Comparison of serum inflammatory factor level between the two groups ($\bar{x}\pm s$, pg/ml).

Groups	Cases	TNF-*α*		IL-6	
		Before intervention	After intervention	Before intervention	After intervention
The study group	48	27.91 ± 7.97	10.22 ± 3.69	22.39 ± 5.79	9.71 ± 1.69
The control group	48	30.02 ± 8.19	16.81 ± 4.15	24.11 ± 6.38	12.51 ± 2.96
*tvalue*		1.279	8.222	1.456	5.691
*Pvalue*		0.204	0.001	0.149	0.001

## Data Availability

We declare that the data was included in our manuscript.
